# Strategies for Advanced Production: A Review of the Use of AI in the Dairy Industry

**DOI:** 10.3390/ani16091363

**Published:** 2026-04-29

**Authors:** Isabela Pérez Núñez, John Quiñones, Gastón Sepúlveda Truan, David Cancino-Baier, Rubén Agregán, José M. Lorenzo, Néstor Sepúlveda, Rommy Díaz

**Affiliations:** 1 Doctorado en Ciencias Agroalimentarias y Medioambiente, Facultad de Ciencias Agropecuarias y Medioambiente, Universidad de La Frontera, Temuco 4780000, Chile; i.perez04@ufromail.cl (I.P.N.); g.sepulveda10@ufromail.cl (G.S.T.); 2 Centro de Tecnología e Innovación en Calidad de la Carne (CTI-Carne), Universidad de La Frontera, Temuco 4780000, Chile; john.quinones@ufrontera.cl (J.Q.); david.cancino@ufrontera.cl (D.C.-B.); nestor.sepulveda@ufrontera.cl (N.S.); 3 Facultad de Ciencias Agropecuarias y Medioambiente, Universidad de La Frontera, Av. Francisco Salazar 01145, Temuco 4811230, Chile; 4 Fundación Centro Tecnolóxico da Carne, Adva. de Galicia n° 4, Parque Tecnolóxico de Galicia, 32900 San Cibrao das Viñas, Ourense, Spain; rubenagregan@ceteca.net (R.A.); jmlorenzo@ceteca.net (J.M.L.); 5 Área de Tecnoloxía dos Alimentos, Facultade de Ciencias, Universidade de Vigo, 32004 Ourense, Ourense, Spain

**Keywords:** artificial intelligence, dairy industry, monitoring, AI-based tools

## Abstract

Artificial intelligence (AI) in the dairy industry is helping to enhance efficiency and sustainability across various key areas, such as animal health, milk quality, milk production analysis, and the monitoring and improvement of dairy products, such as cheese, yogurt, and butter. Beyond productivity, AI-based tools enable companies to measure their environmental impact and forecast the demand for dairy products—factors that are equally critical to this industry. This review aims to address the use of AI from the animal to the final product, within the context of its environmental impact, and to identify areas that require further attention due to their vulnerability in terms of robustness and effectiveness.

## Simple Summary

Artificial intelligence is changing the way milk and dairy products are produced. By using computer systems that can learn and make decisions, dairy farms can better monitor animal health, predict milk production, and maintain higher quality in products such as cheese, yogurt, and butter. These tools also help farmers and companies reduce waste, save energy, and measure their environmental footprint. This article reviews how artificial intelligence is being used from the care of dairy animals to the final processing of milk and its products. This highlights the benefits of these technologies, such as improving efficiency and sustainability, but also points out the need for stronger systems to ensure reliable results. Understanding how artificial intelligence supports the dairy industry can help farmers, scientists, and consumers make better decisions that benefit both food production and the environment.

## 1. Introduction

Artificial intelligence (AI) involves the development of computer systems capable of performing tasks typically requiring human intelligence, such as learning, reasoning, problem-solving, and understanding natural language [1]. It aims to make computers execute functions like those of the human mind, encompassing psychological skills like perception, association, prediction, planning, and motor control [2].

The development of AI began in the mid-20th century, but its progress has accelerated rapidly over the last decade [3]. Initially, AI in natural resource management started with systems for problem-solving and decision-making, which led to the development of other AI procedures, such as integrated systems, intelligent geographic information systems, and animal behavior modeling through AI [4].

Today, AI is highly relevant due to its broad applications across various fields. In business, AI is increasingly used in human resources for employee hiring, retention, and development, as well as for automating tasks like payroll and policy creation [1]. In the food industry, it has been applied to sorting fresh products, managing supply chains, and anticipating consumer preferences [5]. In healthcare, AI is being developed for all aspects of the system, including prevention, treatment, and prognosis prediction, particularly in cardiovascular medicine [3]. Furthermore, AI plays a critical role in firms’ digital transformation projects, supporting existing business operations and creating new opportunities for growth and efficiency [6]. As AI continues to evolve, it presents both challenges and opportunities, especially in reducing the digital divide between developed and developing countries [7].

AI is revolutionizing the dairy industry by improving efficiency and sustainability across multiple key areas [6,8]. One of its most transformative applications is in animal health, where real-time sensor data and advanced analytics are used to monitor animal welfare [9]. These systems facilitate early disease detection [10], personalized feeding adjustments [11], and optimized herd management [12]. Such innovations not only enhance animal well-being but also contribute to increased milk yield and improved milk quality, both essential objectives for productivity [8,13].

In terms of milk quality, AI enables real-time analysis of milk composition [14], detection of contamination [15], and monitoring of nutrient levels. These technologies ensure that milk consistently meets quality standards before being processed into derivatives such as cheese, yogurt, or butter. Furthermore, AI-powered predictive models help producers anticipate peaks and troughs in milk production [16], allowing them to optimize logistics and storage [17], thereby minimizing waste.

AI also plays a pivotal role in the production of dairy derivatives. Advanced tools monitor the maturation process of cheese, optimizing time and conditions to achieve superior quality [18]. Similarly, AI enhances the formulation of yogurt and butter, enabling precise control over texture and flavor to meet diverse consumer preferences [19].

Beyond production, AI empowers dairy companies to measure and mitigate their environmental impact. By analyzing data on water usage, waste management, and carbon footprint, companies can adopt more sustainable practices and improve ecological performance [13]. This is increasingly critical for meeting stringent environmental regulations and addressing growing consumer demand for sustainability [20].

AI’s role extends to market demand management, where advanced predictive tools allow companies to anticipate fluctuations in demand for dairy products with greater accuracy. This enables producers to adjust production and supply chains, reducing losses while ensuring a stable supply that benefits both consumers and the industry as a whole.

This review aims to synthesize the current state of AI applications across the entire dairy production system. We specifically address gaps in the existing literature by providing a detailed examination of AI’s role in demand forecasting and environmental impact assessment, in addition to its more established uses in animal health, milk production, and product quality. The review also critically evaluates the barriers to adoption and discusses future perspectives for the field.

## 2. Artificial Intelligence (AI) in the Dairy Production Industry

AI is revolutionizing the dairy industry by enhancing productivity, improving animal welfare, and addressing environmental challenges. AI applications in dairy farming leverage real-time video analysis, Internet of Things (IoT), and computer vision technologies to monitor cattle identification, body condition assessment, feed intake, and overall animal behavior [9,21]. Machine learning (ML), a subset of AI, focuses on developing algorithms that can learn patterns from data to make predictions or decisions without being explicitly programmed for each task. Its techniques, such as Support Vector Machines (SVM) and decision trees, are fundamental to many modern dairy applications. For instance, ML algorithms can detect subtle behavioral changes that serve as early indicators of diseases such as lameness or mastitis, as well as estrus detection for reproductive efficiency, which is critical for economic success [21,22]. These capabilities allow for timely interventions, ultimately improving animal health, boosting milk production, and ensuring better management practices.

AI-powered systems also contribute significantly to sustainability efforts in the dairy sector. By integrating big data analytics with advanced AI algorithms, these technologies optimize feed efficiency, refine manure management, and improve energy utilization, which directly address greenhouse gas emissions from enteric fermentation and manure processes [13]. Such innovations align with the dairy industry’s pursuit of net-zero emissions, providing benchmarking standards for emissions reduction while fostering transparency across the supply chain [13,23]. Furthermore, AI facilitates predictive analytics for cow health monitoring and farm productivity enhancement, ensuring data-driven decision-making that enhances economic performance and operational efficiency [8].

In precision dairy farming, AI enables near real-time monitoring of cow behavior and health, which promotes early detection of health issues like mastitis and improves body condition assessments, ensuring optimal welfare and productivity [9]. These advancements not only reduce manual, repetitive tasks but also enhance the work environment, allowing farmers to focus on strategic decisions [21]. However, alongside these benefits, ethical considerations arise, including farmer privacy concerns, accountability issues, and the impacts of robotic technologies on animal welfare [24]. Addressing these challenges is essential to fully harness AI’s potential without compromising ethical and operational standards. Despite AI technologies in the dairy industry being predominantly applied to enhance animal welfare; the use of AI for secondary applications, such as analyzing consumer perceptions of dairy products, optimizing the production of dairy derivatives, and assessing broader environmental impacts, remains relatively limited. Expanding AI technologies into these areas could provide valuable insights into market trends, product innovation, and sustainability, further transforming the dairy industry beyond its current operational focus.

AI plays a transformative role in the dairy industry by driving sustainability, improving animal health and welfare, and optimizing farm operations. Through innovations such as precision farming techniques, emissions reduction strategies, and predictive analytics, AI enables a more efficient and environmentally responsible approach to dairy production [13]. As the industry evolves to meet global demand, AI will remain central to addressing economic, environmental, and ethical challenges while ensuring a sustainable future for dairy farming. AI technologies in the dairy industry are predominantly applied to enhance animal welfare; however, the use of AI for secondary applications, such as analyzing consumer perceptions of dairy products, optimizing the production of dairy derivatives, and assessing broader environmental impacts, remains relatively limited. Expanding AI technologies into these areas could provide valuable insights into market trends, product innovation, and sustainability, further transforming the dairy industry beyond its current operational focus. Specific examples of the use of these technologies is shown in Table 1.

**Figure 1:**
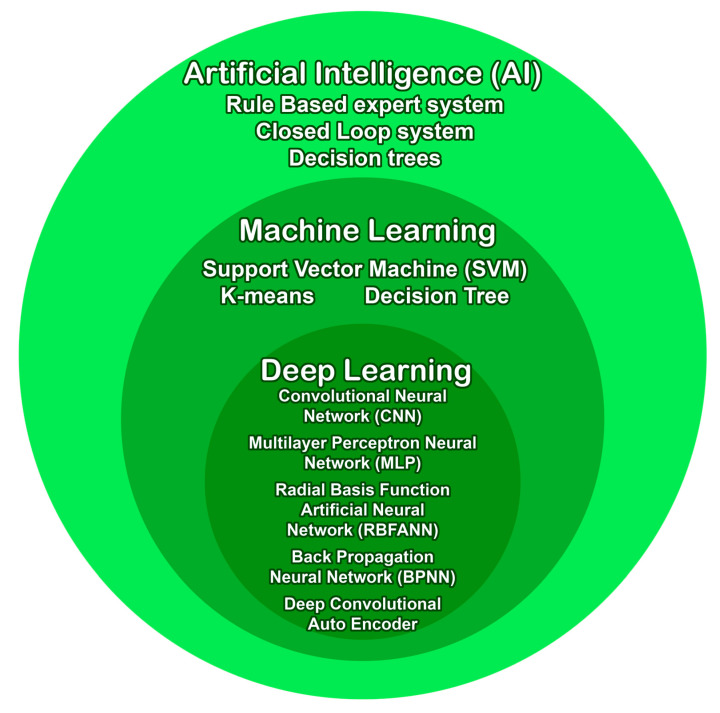
Diagram of artificial intelligence (AI) technologies used in the dairy production industry.

Currently, AI is categorized according to its degree of complexity into three groups: AI, machine learning (ML) and deep learning (DL), with each being more methodologically complex. AI is the broadest category, encompassing various techniques that aim to simulate human intelligence in machines. This includes rule-based systems, expert systems, and fuzzy logic [32]. ML, a subset of AI, focuses on algorithms that can learn from and make predictions or decisions based on data, without being explicitly programmed. ML techniques include support vector machines (SVMs), decision trees, and random forests (RF) [32,33]. DL, the most complex subset, utilizes artificial neural networks (ANN) with multiple layers to extract high-level features from data, enabling more sophisticated pattern recognition and decision-making [34,35]. This progression reflects the increasing sophistication of algorithms and their ability to handle more complex tasks, from rule-based systems to neural networks capable of learning intricate patterns in large datasets. As the field continues to evolve, new hybrid approaches and categorizations may emerge, further refining our understanding of AI and its applications. Some of the AI technologies that have been successfully implemented in the dairy industry are presented in Figure 1.

**Figure 2:**
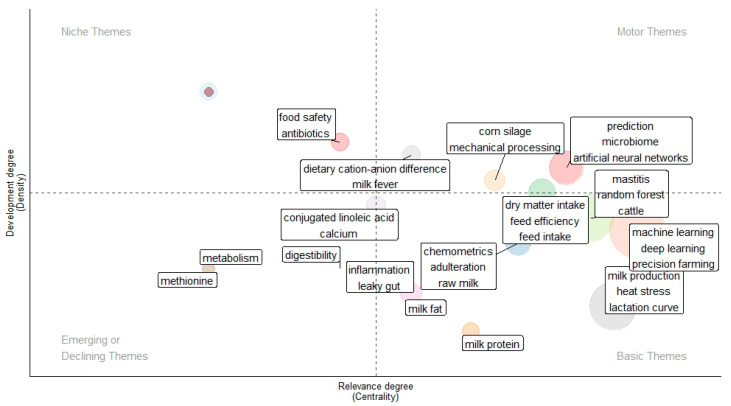
Strategic diagram of research themes related to Artificial Intelligence (AI) in dairy production, based on bibliometric analysis of Web of Science (WoS) and Scopus databases. The *Y*-axis (Relevance Degree/Centrality) represents the importance and interconnection of each theme with other research areas in the field. The *X*-axis (Development Degree/Density) indicates the internal cohesion and maturity of research within each theme. The diagram shows four distinct thematic clusters: Motor Themes (upper-right quadrant) that are both highly relevant and well-developed; Niche Themes (upper-left quadrant) that are specialized but less connected to the broader field; Emerging or Declining Themes (lower-left quadrant) that are underdeveloped and peripheral; and Basic Themes (lower-right quadrant) that are fundamental to the field but require further development.

As AI is constantly developing, core areas such as ML and precision livestock farming emerge as pivotal drivers of innovation, reflecting their significant impact on operational efficiency and animal welfare. Meanwhile, topics like dietary cation–anion difference and lactation curves indicate emerging or declining trends within the field. This analysis underscores the multifaceted role of AI in transforming dairy production, offering insights into both current applications and future research directions. A strategic map of themes related to the use of AI in dairy production, based on a web of science (WoS) and Scopus results, is depicted in Figure 2.

## 3. Artificial Intelligence (AI) for Animal Health and Fitness Estimation

AI has emerged as a powerful tool for improving animal health and fitness estimation, offering numerous applications in veterinary medicine and animal care. AI algorithms have demonstrated impressive capabilities in disease detection and diagnosis, utilizing medical imaging analysis to help veterinarians identify and classify diseases with greater accuracy and efficiency [36]. ML and DL techniques, such as Partial Least Squares Discriminant Analysis (PLS-DA), ANN, and convolutional neural network (CNN) models, have been employed for predicting and characterizing lesions, assisting doctors, veterinarians, and radiologists in their work [36]. AI-driven systems can also analyze vast amounts of data from electronic health records and genetic profiles to recognize trends and predict disease outbreaks, enabling remote monitoring of vital signs and facilitating swift preventative measures and personalized treatments [36,37]. Interestingly, AI is not only enhancing diagnostic capabilities but also revolutionizing animal nutrition through innovative algorithms and data analytics [38]. Moreover, AI technologies are being used to understand animal behaviors and emotions, providing deeper insights into their well-being and sources of stress [38]. This holistic approach to animal health assessment goes beyond traditional methods, offering a more comprehensive understanding of animal welfare. As such, AI is transforming animal healthcare by improving disease detection, enhancing diagnostic accuracy, and enabling personalized treatment plans. From early disease detection to continuous monitoring and surveillance, AI offers promising solutions for proactive disease management in animals [37]. As the field continues to evolve, addressing ethical considerations, data privacy concerns, and regulatory frameworks will be crucial for the responsible and effective application of AI in animal healthcare [36,37].

Recent advancements in AI have revolutionized various aspects of livestock management, with dairy farming standing at the forefront of this transformation. AI applications have enabled more precise and efficient approaches to managing health and reproduction, significantly enhancing both operational efficiency and animal welfare.

In dairy cattle research, Monteiro et al. [16] applied AI to predict microbial community compositions associated with specific phenotypic traits in cows. Their study employed a differential abundance analysis (DAA) method, integrating engineered variables into a linear regression model to analyze microbial data comprehensively. This approach identified key microbial taxa exhibiting consistent differences across analytical models, facilitating a better understanding of microbiota–trait associations.

The ThinkDairy Data-Mining Hub, developed by Nogoy et al. [25], is an innovative AI-driven tool that uses IoT-generated data to monitor dairy cow health in real time. By integrating diverse data sources and employing ML algorithms like K-Nearest Neighbors (KNN), the hub accurately detects conditions such as heat stress and subclinical mastitis. This approach significantly improves detection accuracy, reduces misclassification, and enhances decision-making in farm management. The study also highlights future research directions, including exploring advanced algorithms to refine detection capabilities further, emphasizing AI’s transformative role in modernizing and improving dairy farming practices.

Health monitoring has seen remarkable progress with the integration of AI technologies. Shi et al. [10] explored the potential of wearable sensors combined with RF algorithms to monitor the health status of dairy cattle. By incorporating Explainable AI (XAI) techniques such as SHAP, their study elucidated behavioral patterns that served as early indicators of mastitis. These findings not only improved disease detection accuracy but also enabled farmers to take preventive actions promptly. Other studies have demonstrated the utility of ANN and RF models in identifying bovine mastitis by analyzing somatic cell count, lactose concentration, and electrical conductivity data [39]. The deployment of such models has proven effective in distinguishing between subclinical and clinical mastitis, addressing a major concern in dairy production. Non-invasive body sensors further enhance disease monitoring, as evidenced by ANN applications that detect behavioral anomalies associated with health issues in real time, achieving impressive diagnostic accuracy [40].

Reproductive management has also benefited substantially from AI innovations. Nagahara et al. [41] introduced a DL model, EfficientNET, to predict the optimal timing for artificial insemination by analyzing uterine images. This model achieved improved accuracy in pregnancy diagnostics compared to traditional methods, offering a non-invasive solution to optimize reproductive outcomes. Moreover, ML algorithms, such as decision trees, SVM, and ANN, have been applied to multimodal sensor data to detect estrus. These systems combine physiological indicators with behavioral observations, resulting in enhanced sensitivity and precision [42,43]. Automated estrus detection using fuzzy logic has also shown promise in reducing false positives, further streamlining reproductive efficiency [44].

In automated operations, AI has been pivotal in addressing mobility-related issues in livestock. For instance, systems like CattleEye utilize ML and computer vision to assess cattle mobility by analyzing limb movement patterns. This technology, according to the authors “outperforms the human scorer in the detection of severe lesions when sensitivity is concerned [45] due to the innate advantages of automatic systems and the frequency of scoring”, offering consistent assessments across farms and contributing to better lameness management [45]. Similarly, body condition scoring (BCS), a critical indicator of animal health, has been revolutionized by convolutional neural networks (CNNs). These networks analyze top-down images of cows, providing real-time BCS estimations that are comparable to manual scoring methods [46]. Extending this technology to other species, Temenos et al. [47] developed a lightweight CNN model for pose-independent BCS in goats using low-cost cameras, demonstrating remarkable accuracy and scalability for broader agricultural applications.

AI has streamlined livestock management by automating estrus detection and cow identification. DL models like CNNs and YOLOv5 improved estrus detection accuracy and reduced reliance on error-prone manual methods [48]. Zhao et al. [27] utilized infrared thermography in combination with YOLOv8 models to detect respiratory rates in dairy cows, achieving an accuracy exceeding 94%. This non-invasive approach represents a significant advancement in early respiratory disease detection. Furthermore, DL models such as 1D-CNNs and long short-term memory (LSTM) networks have enabled precise classification of cattle behaviors, including grazing, walking, and standing. These models enhance understanding of behavioral patterns, promoting better welfare and productivity [49]. AI applications in calving management have also shown promise, with systems monitoring temperature fluctuations at the ventral tail base to predict parturition timing. These tools provide continuous, non-invasive monitoring, reducing risks associated with delayed interventions [50].

Assisted reproductive technologies (ART) have been optimized through AI advancements. Mikkola et al. [51] highlighted the role of AI in improving embryo quality assessment and fertility evaluation. Despite challenges such as remote data connectivity, these technologies have demonstrated significant potential in enhancing reproductive outcomes. Transformer models, a cutting-edge AI approach, have advanced pregnancy loss prediction in dairy cows by integrating large datasets with high accuracy and interpretability [52]. In addition, Bauer and Jagusiak [28] applied multilayer perceptron (MLP) networks to detect subclinical ketosis in dairy cows, showcasing the effectiveness of neural networks in managing herd health. Kurras and Jakob et al. [53] utilized 360-degree cameras to monitor dairy cow behaviors in free-stall barns, highlighting clear diurnal activity patterns. Despite challenges like occlusion, this non-invasive system shows promise for enhancing welfare and health management.

Disease prediction systems have made significant strides in early detection, crucial for maintaining herd health. As summarized in Table 2, these AI technologies have demonstrated measurable improvements in detection accuracy and early warning capabilities for various health conditions. For example, LSTM models analyzing rumination and activity data achieved exceptional accuracy in predicting anaplasmosis onset, even days before clinical symptoms appeared [26]. These advancements underscore the importance of continuous monitoring for timely and effective interventions.

Artificial intelligence applications in animal health have evolved from early diagnostic systems to fully integrated predictive frameworks. Classical algorithms such as Random Forest (RF) and Support Vector Machines (SVM) were initially used for classification of visible disorders, such as lameness and mastitis [10], while modern research focuses on deep learning models that exploit multimodal sensor data to predict disease before clinical onset [26]. The core contribution of this subfield lies in the transition from reactive to predictive veterinary practice.

A second line of progress involves the adoption of non-invasive monitoring systems. Whereas early approaches relied on wearable accelerometers and thermographic sensors, recent studies employ convolutional and sequential architectures for automatic behavior recognition through computer vision [27]. These methods reduce stress in dairy cows while maintaining accuracy comparable to human observation.

Nevertheless, the gap between controlled-environment accuracy and practical deployment remains significant. Data transmission constraints and farm-specific variability limit generalization, suggesting the need for domain-adaptive or federated models to bridge laboratory and field conditions.

Overall, these studies highlight AI’s transformative potential across various facets of dairy farming, such as health, reproduction, and welfare. However, challenges such as data quality, connectivity, and algorithm interpretability remain, emphasizing the need for ongoing research and innovation to fully realize the benefits of AI in livestock management. Collectively, these studies demonstrate AI’s transformative potential in proactive animal health management, shifting the industry from treatment to prevention. However, the reliance on high-quality, continuous data streams and the ‘black box’ nature of many complex algorithms present significant barriers to practical, on-farm implementation. Future work must focus on developing more robust, explainable, and integrated systems that farmers can trust and use effectively.

## 4. Artificial Intelligence (AI) for Dairy Production Yield

AI has revolutionized milk production monitoring in the dairy industry, delivering potential benefits to farmers by improving efficiency and decision-making processes. Through ML algorithms and computer vision techniques, AI enables the monitoring of various aspects of dairy cattle health and behavior, ultimately enhancing milk yield and quality.

One of the primary applications of AI in dairy farming involves the use of contact sensors, vision analysis, and ML technologies. These tools allow near-real-time tracking of cow behavior, health, and management practices. For example, AI-powered systems can promptly detect conditions such as mastitis [9], evaluate body condition scores, and measure feed intake with high precision. Furthermore, biometric identification systems facilitate individual animal tracking, enabling continuous monitoring and personalized care [56]. Early detection of diseases in cows will allow for urgent halts in milk production, saving production costs and safeguarding animal health. AI applications extend to predicting milk production based on environmental and physiological factors, where models like Back Propagation Neural Networks (BPNN) optimized with GA have successfully forecasted milk yield by analyzing parameters such as relative humidity, heart rate, environmental temperature, and cow body temperature. Thus, studies employing BPNN optimized with GA have demonstrated the ability to forecast milk yield based on the afore mentioned parameters. These insights help farmers make data-driven decisions to optimize production.

AI-driven ML algorithms have been leveraged to predict lactation curve characteristics in dairy sheep, enabling precise modeling of total milk yield, peak yield, and time to peak yield through the analysis of extensive datasets [57]. These models provide insights that can guide breeding and nutritional strategies to optimize milk production. Similarly, Siachos et al. [11] explored AI applications in automated dairy cattle lameness detection. Their work employed regression tree models to predict the impact of dietary interventions on milk composition and yield. By integrating feed intake data with physiological parameters, the study demonstrated how AI could identify optimal feeding strategies, significantly improve resource utilization while maintaining milk quality.

In milk pasteurization, Hadi et al. [58] explored the potential of artificial intelligence of things (AIoT) to enhance efficiency and product quality. By integrating the C4.5 algorithm into a low-temperature, low-time pasteurization system, the study introduced a fully automated process that adjusts temperature and duration based on real-time data. The system utilizes temperature and pH sensors to feed the algorithm, categorizing milk quality as “good”, “medium”, or “poor”. After local training, the parameters are uploaded to a Platform as a Service (PaaS) framework, facilitating scalable and accurate decision-making. This approach minimizes human error, reduces costs, and ensures consistent product quality.

Logistical challenges in milk distribution, such as those faced in Morocco, have also benefited from AI. Karouani et al. [17] implemented the Ant Colony Optimization (ACO) strategy combined with Internet of Things (IoT) technologies to optimize milk collection routes. Sensors monitor milk quantities at farms, while ACO algorithms determine the most efficient paths for truck routing, considering time, truck space, and fuel consumption. This system improves route planning, reduces the number of trucks needed, and enhances connectivity between farms and transportation networks, resulting in more sustainable logistics.

In Turkey, Oztuna Taner et al. [20] addressed productivity and environmental impacts in a dairy factory through a MLP model based on ANN. Using nine product types as input parameters, the model was trained with the Levenberg–Marquardt algorithm to predict factory productivity with exceptional accuracy (MSE: 4.02 × 10^−6^, R^2^: 0.99984). Optimized with 13 neurons in the hidden layer and activation functions such as Tan-Sig and Purelin, the model enabled precise analysis of production efficiency, contributing to extended shelf-life and reduced environmental impact.

Research in dairy yield prediction integrates physiological and environmental data through neural networks and hybrid optimization models. Early studies demonstrated the potential of Backpropagation Neural Networks (BPNN) for milk yield estimation [16]. However, accuracy improvements emerged when meta-heuristic algorithms such as Ant Colony Optimization (ACO) or Particle Swarm Optimization (PSO) were embedded to optimize hyperparameters [17].

The fundamental contribution of this research area lies in establishing the link between animal-level prediction and farm-scale optimization. Models that estimate yield per cow can be connected to route-planning algorithms for milk collection, allowing simultaneous biological and logistical efficiency.

The integration of AI in the dairy industry spans diverse applications, from monitoring cattle health to optimizing pasteurization and distribution systems. These advancements highlight the transformative potential of AI in improving productivity, reducing environmental footprints, and ensuring high-quality dairy products. Detailed information on improvements made and possible applications of AI models in the dairy industry are detailed in Table 3.

## 5. Artificial Intelligence (AI) for Milk Quality and Safety

Beyond production monitoring, AI technologies play a crucial role in enhancing milk quality and ensuring product safety. Edge-AI-based architectures, for example, implement in situ milk adulteration detection techniques, providing real-time monitoring of milk quality. A CNN model deployed on an edge device (Jetson Nano) achieved 94.87% accuracy in classifying adulterants in milk using Fourier Transformed Infrared (FTIR) data [15]. Similarly, Adaptive Neuro-Fuzzy Inference Systems (ANFIS) predict milk moisture and fat contents using color components, enabling accurate real-time quality assessments for both conventionally and non-thermally pasteurized milk [14]. ANN further enhance quality control by predicting the electrical conductivity of raw and pasteurized milk based on electric field strength and mass flow rate, facilitating online assessment of milk’s physical attributes [62]. These AI-driven technologies not only improve quality control through automated inspection systems capable of detecting microscopic defects but also ensure compliance with regulatory standards by providing real-time monitoring of manufacturing processes [63]. Consequently, AI applications contribute to more efficient, accurate, and sustainable milk quality monitoring, enhancing food security and production sustainability [14,62].

AI is making significant progress in lactose monitoring and enzymatic processes in dairy production. Dos Santos et al. [64] evaluated lactose hydrolysis using β-galactosidase enzymes and demonstrated that AI-based predictive algorithms, such as SVM, can optimize lactose monitoring by accurately predicting lactose levels based on cryoscopy values and glucose concentrations. This approach minimized measurement errors, achieving a mean difference of only 0.001 between observed and predicted lactose values, thereby enhancing the reliability of lactose measurement in dairy production. Furthermore, AI facilitates the integration of cryoscopy and enzymatic kits for comprehensive lactose analysis, supporting stringent quality control and ensuring products meet regulatory standards for lactose content.

Advanced AI models, including Hybrid Blended Deep Learning (HyBDL) and combinations of Gated Recurrent Units (GRU) with Residual Networks (ResNet), have been employed to overcome challenges in Milk Quality Analysis (MQA). Mhapsekar et al. [29] highlighted that HyBDL, which combines CNNs and Recurrent Neural Networks (RNNs), achieved an impressive accuracy of 98.03% with lower Mean Squared Error (MSE) scores, outperforming traditional DL and ML models. Similarly, Tolba et al. [65] utilized a GRU-ResNet model to predict milk quality traits with high accuracy (R^2^ = 0.95, RMSE = 0.22), effectively addressing issues such as the vanishing gradient problem and overfitting in DL models. These studies underscore the importance of hybrid DL models in enhancing the accuracy and robustness of milk quality predictions, enabling more reliable and efficient dairy farming practices.

AI also extends to the detection of key components in dairy products, as demonstrated by Yüksek et al. [66], who developed a method to determine vitamin B1 levels using a combination of natural deep eutectic solvent-based sonication-assisted liquid-phase microextraction (NDES-SA-LPME) and UV-Vis spectrophotometry. By employing AI techniques such as Radial Basis Function Artificial Neural Networks (RBF-ANN) and Gaussian Process Regression (GPR) with an Exponential kernel, the study achieved high accuracy in predicting recovery rates and optimizing extraction efficiency. This integration of AI into analytical methods provides faster, more efficient, and cost-effective solutions for evaluating nutritional components, marking a significant advancement in dairy product analysis.

Finally, AI enhances quality monitoring and traceability in dairy production chains through innovative systems like BEST, as demonstrated by Dragone et al. [30]. This sensor-driven system employs supervised MLP neural networks and unsupervised neural network approaches to monitor quality and traceability, identifying specific cows with unique characteristics and ensuring comprehensive herd health management. The AI component of BEST effectively categorizes milk samples, minimizing false negatives and positives, and supports HACCP (Hazard Analysis and Critical Control Points)-like protocols to prevent milk-borne zoonoses and toxicological risks. This patented system underscores AI’s role in enabling environmentally sustainable and cost-effective quality monitoring, enhancing food safety from farm to fork. The quantitative performance metrics of these AI-driven quality control systems, including detection accuracy rates, are compiled in Table 2.

The application of AI for milk quality control reflects the transition from laboratory-based to real-time, non-destructive assessment. Early Adaptive Neuro-Fuzzy Inference Systems (ANFIS) allowed prediction of physicochemical components [62], paving the way for Convolutional Neural Networks (CNN) to identify adulteration patterns in spectroscopic data [15]. Hybrid deep-learning models, such as HyBDL and GRU-ResNet, further enhanced sensitivity and specificity in detecting bacterial contamination [29,65].

These applications not only improve operational efficiency and product quality but also contribute to sustainable milk production and increased food security, highlighting the transformative potential of AI in modern dairy farming. Specific examples, their accuracy and contribution to the field are specified in Table 4.

## 6. Artificial Intelligence (AI) for Improving the Quality and Sensory Experience of Dairy Products

AI is transforming the dairy industry by enhancing efficiency, transparency, and quality throughout the supply chain. The integration of AI-driven systems, combined with technologies like blockchain and Radio Frequency Identification (RFID), enables precise tracking, real-time updates, and verification of dairy products. Blockchain records provide secure, tamper-proof insights into supply chain operations, while RFID tagging allows continuous monitoring at each stage of processing and distribution. This combination ensures product authenticity and safety while streamlining operations through data-driven insights [69,70]. AI also plays a critical role in evaluating manufacturing data stored on blockchain systems, facilitating proactive maintenance and consistent quality control during production [70]. However, implementing these technologies poses significant challenges, such as cybersecurity threats, organizational transformation, and operational barriers, which must be addressed for successful adoption [71].

Beyond supply chain management, AI is making strides in product innovation and consumer-focused research. For instance, Gülsen et al. (2023) [31] examined the emotional responses of infants aged 6–11 months to various food groups, including dairy products like yogurt and cheese. Using OpenFace, a tool based on the Facial Action Coding System (FACS), they analyzed facial expressions to assess emotions such as surprise, sadness, and disgust. Notable findings included stronger negative reactions, such as disgust and fear, from breastfed infants compared to non-breastfed ones, underscoring the importance of feeding history in product development. This research highlights the potential for AI to guide the dairy industry in creating sensory-aligned products while also educating caregivers on optimal feeding practices.

AI is also revolutionizing cheese production, particularly in monitoring ripening and ensuring consistent quality. Loddo et al. (2022) introduced an AI-driven system using computer vision and ML to automate cheese ripening assessment [72]. By analyzing images of Pecorino cheese at various stages of ripeness, their model achieved a 98% classification accuracy, offering a reliable and non-invasive alternative to traditional methods. This innovation enables real-time monitoring and improves quality control processes. Building on these advancements, Loddo et al. (2025) proposed an AI-based method for determining curd cutting time during cheese production, addressing variability issues often faced by small-scale producers [73]. Their system combines image analysis with temporal sequence data, allowing immediate adjustments and enhancing product consistency and efficiency. Similarly, Zedda et al. (2024) introduced a hybrid ML framework that integrates deep and shallow learning techniques to automate cheese ripeness detection [18]. Using a novel dataset of cheese wheel images, their method achieved an F-measure score of 0.991, demonstrating the potential of AI to streamline quality assessment and production processes.

In addition to production efficiency, AI contributes to improving sensory evaluation and consumer engagement. Bi et al. (2022) employed a hybrid optimization algorithm combining GA, particle swarm optimization, and tabu search to refine sensory attributes of yogurt [19]. This method, which incorporates deep convolutional autoencoders and support vector machines, identifies consumer-preferred sensory features, enabling the development of products that align with market expectations. The study also emphasizes the potential for applying these technologies across various industries. Furthermore, Tzafilkou et al. (2023) explored the use of AI tools like FaceReader Online™ to analyze consumer micro-expressions during social media advertisements for food products [74]. Their findings demonstrated that Neural Networks could predict purchase intent with 90–91% accuracy, highlighting the value of emotion detection in optimizing marketing strategies. Table 2 provides specific performance data for these AI applications in dairy product quality assessment and optimization.

AI is redefining the sensory evaluation and quality of dairy products, shifting it from subjective human panels to quantitative, objective, and automated methods. The main contribution of these studies is the creation of a direct bridge between manufacturing process data and final consumer perception/acceptance. There are two main streams of research that are beginning to relate: one focuses on objective product optimization (such as cheese ripening [18,72] and yogurt attributes [19], and the other on direct assessment of consumer response [31,74]. The trend towards using computer vision for tasks that previously required a human expert (e.g., determining curd cutting time [72]) represents a crucial advance for standardization and scalability in the industry. The next evolutionary step will be to integrate these two streams, using consumer sensory data to feedback and automatically adjust production parameters. Overall, AI is proving to be a transformative force in the dairy industry, enhancing supply chain transparency, product quality, and consumer satisfaction. By leveraging advanced technologies such as blockchain, computer vision, and optimization algorithms, the industry can address longstanding challenges while embracing innovative opportunities. However, the successful implementation of AI requires addressing strategic and technical barriers to ensure widespread adoption and sustained impact. Table 5 indicates specific examples and metrics that have been considered for these mean.

## 7. Artificial Intelligence (AI) Based Dairy Databases

AI-based dairy databases are emerging as transformative tools in the dairy industry, offering significant advancements across various facets of farming and production. These systems, powered by AI and ML algorithms, integrate data from diverse sources such as activity monitors, dairy herd improvement records, herd management systems, and milking logs, thereby enabling comprehensive data-driven decision-making [79]. This integration not only enhances management efficiency and optimizes economic performance but also promotes animal welfare, contributing to the overall sustainability of dairy operations. These databases are particularly impactful in addressing environmental challenges, facilitating the development of benchmarking standards for emissions, and advancing the industry’s goals toward net-zero emissions [13]. By tracking emissions across Scope 1, 2, and 3 levels, they foster transparency and collaboration throughout the supply chain [23]. Furthermore, AI-powered predictive analytics enhance feed efficiency, manure management, and energy use, reducing the environmental footprint of dairy farming and empowering farmers to remain competitive while meeting global sustainability challenges [13].

A key innovation in this field is CowMesh [80], introduced by Pakrashi et al. (2023), which provides a novel data mesh architecture designed to unify disparate data sources within the dairy sector. This architecture enhances prediction and monitoring capabilities, improving decision-making and operational efficiency. By integrating diverse datasets, CowMesh supports the development of advanced predictive models and monitoring systems that provide stakeholders with actionable insights. Its semantic layer ensures interoperability among data sources while maintaining privacy and governance through standardized data-sharing policies. CowMesh’s decentralized approach allows for independent operation of data products, fostering transparency and trust among stakeholders [80]. ML techniques embedded within the system enable early detection of health issues such as mastitis and ketosis, reducing economic losses. Moreover, real-world applications of CowMesh in Irish dairy farms demonstrate its potential to address data fragmentation and deliver economic benefits to farmers. Future research on governance frameworks and semantic layer customization aims to further explore its scalability and adaptability across agricultural contexts [80].

AI’s potential extends beyond data integration to specific applications such as lameness detection in dairy cows. Lameness, a prevalent and costly health issue, has traditionally been diagnosed through subjective visual assessments by veterinary clinicians. To address these limitations, Ismail et al. (2024) introduced CowScreeningDB, a publicly accessible multi-sensor database [81]. This database includes inertial measurement unit (IMU) data collected from 43 cows and integrates it with clinician-assessed lameness scores, achieving an average classification accuracy of 77% in detecting lame cows. By adhering to FAIR principles, CowScreeningDB ensures transparency, quality, and reusability, fostering reproducible research and innovation. Additionally, DL models leveraging this database demonstrate the potential for real-time lameness detection, paving the way for handheld monitoring devices to improve operational efficiency and animal welfare [81].

In addition to health monitoring, AI is enhancing livestock activity tracking through innovative IoT devices. Dutta et al. (2022) developed the MOOnitor, a neck-mounted device equipped with a temperature sensor, GPS module, and 3-axis accelerometer to continuously monitor parameters like body temperature, movement, and walking speed [82]. Using advanced ML algorithms such as eXtreme Gradient Boosting (XGBoost) and RF, the MOOnitor classifies activities like standing, lying, walking, and ruminating with 97% accuracy. This real-time activity tracking supports early detection of health issues such as estrous, mastitis, and foot-and-mouth disease, setting a benchmark in precision livestock management. By integrating multi-sensory data and advanced AI techniques, the MOOnitor contributes significantly to sustainable and data-driven livestock management [82].

The application of AI in assessing the environmental impact of dairy is in an emerging phase, focused on optimizing specific processes rather than holistic assessment. The main contribution of these studies is to provide data-driven tools to quantify and reduce emissions at critical points in the value chain, such as manure management [83] and refrigeration energy efficiency [84]. The relationship within the literature presented reveals a fragmented approach: predictive ML models address specific, localized problems (manure temperature, equipment faults), while conceptual frameworks [13,85] advocate for the full integration of Industry 4.0 technologies. This disconnect underscores the most significant gap in this field: the lack of comprehensive AI systems that can model and optimize the environmental footprint of an entire dairy operation or supply chain in a unified, rather than isolated, manner. Compared to animal-level applications, environmental AI tools show great promise but require further development to achieve similar levels of integration and predictive accuracy at scale. AI-driven tools such as dairy databases, predictive systems, and IoT devices are reshaping the dairy industry. By integrating advanced analytics, improving operational efficiency, and addressing health and environmental challenges, these innovations are building a more sustainable and resilient future for the sector. Further examples of these technologies being implemented for databases and data integration can be found in Table 6.

## 8. Artificial Intelligence (AI) for the Estimation of the Environmental Impact of Dairy Products

The convergence of AI and Big Data technologies is driving transformative change in the dairy industry, offering innovative tools to enhance environmental sustainability and operational efficiency. These technologies are pivotal in optimizing feed utilization, refining manure management, and improving energy efficiency, all of which contribute to reducing greenhouse gas emissions [13]. At the same time, AI enables predictive analytics for feed optimization, real-time monitoring through sensor networks, and precision farming practices that align with international climate commitments while addressing rising global milk demand [13,87]

Radun et al. [87] highlight AI’s capacity to manage and analyze large datasets, enabling precise decision-making essential for modern dairy farming. By forecasting optimal practices and monitoring attributes like animal health and production efficiency, AI enhances animal welfare while mitigating environmental impacts, such as ammonium pollution linked to intensive dairy operations. These insights align closely with the findings of Lee et al. [84], whose application of fault detection and diagnosis (FDD) systems in refrigeration demonstrates AI’s ability to improve energy efficiency, reduce costs, and address data scarcity challenges through advanced techniques like auto-scale transfer learning. This synergy between AI-driven operational enhancements and sustainability objectives underscores the role of technology in reducing the environmental footprint of dairy production.

The integration of Industry 4.0 technologies, as explored by Hassoun et al. [85], further amplifies these benefits. Smart sensors, IoT, robotics, and blockchain enhance traceability, optimize supply chains, and reduce production waste. These advancements not only improve product quality and operational resilience but also create a roadmap for “Dairy 4.0”, where resource efficiency and environmental sustainability coexist. AI’s applications, such as predicting consumer demand, detecting food adulteration, and forecasting spoilage, extend the industry’s capabilities while minimizing waste and enhancing profitability.

Świderski et al. [88] provide additional perspective by demonstrating how ANN can optimize transport systems, an often-overlooked aspect of the dairy supply chain. Efficient logistics reduce operational costs and emissions, directly supporting the broader sustainability goals of the industry. The predictive capabilities of ANNs, combined with real-time assessments, mirror AI’s potential in precision dairy farming, where it can inform feeding strategies, monitor environmental impacts, and support sustainable production [87].

At the farm level, Genedy et al. [83] emphasize the importance of ML in addressing specific challenges such as manure management. Their study demonstrates how ML algorithms improve the accuracy of manure temperature predictions, a critical factor in nutrient cycling and emissions reduction. By integrating diverse datasets, including weather variables and storage system characteristics, the research illustrates the value of advanced analytics in creating sustainable manure management practices. These findings complement the broader potential of AI in enabling precise life cycle assessments and aligning dairy practices with climate commitments [13,83]. Further examples of the implementation of AI in this context can be found in Table 7.

While the potential of AI in the dairy industry is immense, challenges remain. The energy consumption and carbon footprint of AI systems themselves present a paradox that requires careful management. Research into low-energy, environmentally sustainable AI systems is critical to achieving long-term benefits [87,89]. Moreover, successful implementation demands addressing issues such as data security, stakeholder collaboration, and the accessibility of these technologies to ensure widespread adoption [85,89].

The application of AI in assessing the environmental impact of dairy is in an emerging phase, focused on optimizing specific processes rather than holistic assessment. The main contribution of these studies is to provide data-driven tools to quantify and reduce emissions at critical points in the value chain, such as manure management [83] and refrigeration energy efficiency [84]. The relationship within the literature presented reveals a fragmented approach: predictive ML models address specific, localized problems (manure temperature, equipment faults), while conceptual frameworks [13,85] advocate for the full integration of Industry 4.0 technologies. This disconnect underscores the most significant gap in this field: the lack of comprehensive AI systems that can model and optimize the environmental footprint of an entire dairy operation or supply chain in a unified, rather than isolated, manner. Compared to animal-level applications, environmental AI tools show great promise but require further development to achieve similar levels of integration and predictive accuracy at scale. By intertwining advancements in precision farming, supply chain optimization, and sustainable operations, AI offers a comprehensive solution to the dairy industry’s most pressing challenges. Interdisciplinary collaboration, as noted across studies, will be crucial to fully realize this potential, ensuring that AI-driven innovations translate into measurable environmental and economic benefits. These collective efforts, ranging from improving operational efficiency to reducing emissions, chart a path toward a sustainable and resilient future for dairy production.

## 9. Artificial Intelligence (AI) for Demand Prediction of Dairy Products

The application of AI to anticipate dairy demand data is particularly attractive, as it may significantly enhance profitability and outperform traditional methods in terms of accuracy and efficiency. Recent advancements underscore the integration of AI methodologies, statistical tests, and meta-heuristic algorithms, setting new benchmarks for predictive accuracy and practical utility.

Artificial intelligence methods are demonstrably viable and effective for dairy product demand prediction, with studies spanning national and regional implementations across multiple countries. Research examining AI applications including neural networks, support vector regression, and deep learning architectures alongside traditional statistical methods has shown consistent success across diverse dairy markets [90,91]. The evidence confirms that multiple AI approaches are suitable for dairy demand forecasting, with method selection depending on demand characteristics, temporal resolution, and geographic scale rather than universal algorithm superiority.

As an example of earlier works in the use of AI in this area, Doganis et al. [92] focused on sales forecasting for short shelf-life food products, proposing a hybrid methodology combining Radial Basis Function (RBF) neural networks with genetic algorithms (GA). The RBF networks modeled complex, non-linear relationships, while the GA optimized input variable selection, addressing challenges such as redundancy and variable correlations. Applied to real sales data from a dairy manufacturer, the GA-RBF framework outperformed traditional methods, underscoring its potential to improve forecasting accuracy and operational efficiency.

The effectiveness of different AI approaches varies significantly by application context. Comparative analyses reveal that certain models excel in specific demand environments, with performance depending on seasonal patterns, market stability, and data granularity [90]. This context-dependent performance underscores the importance of matching methodological approach to specific demand characteristics rather than seeking a universally superior algorithm.

In more recent examples, Goli et al. [93] introduced a pioneering framework combining statistical tests, time-series neural networks, and enhanced multi-layer perceptron (MLP) neural networks. This framework, tailored for forecasting dairy product demand (DPD) in Iran, utilized a robust selection process to identify significant economic and social indicators, filtering them with Pearson correlation coefficients. The study employed advanced meta-heuristic algorithms, including Gray Wolf Optimization (GWO) and Cultural Algorithm (CA), to optimize MLP performance. Historical data from 2013 to 2017 were used to train and validate the model, achieving a coefficient of determination of 71.9%. This hybrid approach reduced forecast errors by 1.8 times compared to traditional methods, illustrating the effectiveness of integrating statistical analysis with AI tools to model complex and non-linear relationships.

Critical success factors for AI implementation in dairy demand forecasting include systematic feature selection using statistical methods. Research indicates that different predictor variables emerge as dominant across market scales, with macroeconomic factors prevailing in national markets while infrastructure variables prove more relevant for regional implementations [91]. The finding that temporal resolution significantly impacts prediction accuracy further highlights how data aggregation approaches must be carefully matched to forecasting objectives.

Building on this, Goli et al. [54] enhanced their methodology by incorporating the Runner-Root Algorithm (RRA) into an improved framework that combined MLP, ANFIS, and LSTM networks. The integration of RRA significantly increased predictive accuracy, with the MLP-RRA model achieving a coefficient of determination of 98.19%. This study highlighted the superiority of hybrid models, demonstrating that the inclusion of advanced optimization techniques improves demand forecasting for perishable products. The practical implications are vast, enabling better production planning, inventory management, and resource allocation while minimizing waste in the dairy supply chain.

Goli et al. [55] extended these innovations by integrating Support Vector Regression (SVR) with MLP and ANFIS models, enhanced by meta-heuristic algorithms such as Invasive Weed Optimization (IWO) and Particle Swarm Optimization (PSO). Their comprehensive framework not only improved predictive accuracy but also addressed the limitations of conventional regression methods, which often fail to capture the complexities of DPD. By leveraging time series neural networks to account for historical trends and incorporating relevant socio-economic variables, the study further solidified the role of AI in enhancing forecasting reliability. These findings underscore the transformative potential of hybrid AI methodologies in the dairy industry and beyond.

In a broader context, ML algorithms like RF have also proven effective in demand forecasting across various sectors [94]. For the dairy industry, AI enables the analysis of diverse factors, including economic indicators, weather conditions, and historical consumption patterns, to deliver precise forecasts. By optimizing supply chain management and reducing waste from products with short shelf lives, these technologies enhance overall efficiency [55,95]. Furthermore, traditional economic models, such as the two-stage budgeting procedure and the complete demand system, remain relevant by incorporating demographic effects into demand estimation [96]. The interplay between these approaches and AI-driven methods suggests that combining their strengths could yield more comprehensive and robust forecasts.

Finally, in Latin America, da Silva et al. [97] explored sentiment analysis for dairy products in Brazil, creating a balanced dataset of tweets to train models for classifying consumer sentiment. By integrating multiple natural language processing (NLP) tools and applying data balancing techniques like Synthetic Minority Oversampling Technique (SMOTE), the study demonstrated the importance of addressing class imbalances in training datasets. This approach improved model performance, providing valuable insights into consumer behavior and enabling businesses to adapt their strategies effectively. The use of AI-driven sentiment analysis, enriched with subfields such as computer vision and data mining, further enhanced real-time monitoring and decision-making capabilities in the dairy industry. The substantial improvements in forecasting accuracy achieved by these AI methodologies are quantitatively documented in Table 2.

The forecasting of dairy product demand using AI has undergone a marked evolution towards hybrid and metaheuristic models to capture the complexity of perishable markets. The fundamental contribution of this line of research is overcoming the limitations of traditional statistical models, achieving unprecedented accuracy (R^2^ > 0.98) [54] that enables waste reduction and production optimization. The relationship between the studies shows a clear hierarchy of complexity: base models like MLP or SVR significantly improve their performance when optimized by algorithms like RRA or IWO [54,55]. This trend towards hybridization demonstrates that combining the learning capacity of neural networks with the global search power of metaheuristic optimizers is the most effective path for this problem. An emerging gap that these quantitative forecasting studies do not fully address is the integration of real-time qualitative data, such as sentiment analysis [97], to capture sudden demand shocks driven by market factors or social media. While animal health applications focus on real-time monitoring, demand forecasting excels at strategic planning, representing complementary AI applications across the dairy value chain. These studies, among further studies shown in Table 8 collectively highlight the transformative impact of AI and hybrid methodologies on demand forecasting in the dairy industry. By addressing the unique challenges of perishable products, such as short consumption periods and market volatility, these approaches provide actionable insights for improving supply chain management and resource allocation. The integration of statistical tests, advanced neural networks, and meta-heuristic algorithms represents a significant advancement, bridging the gap between theoretical innovation and practical application. Future research should continue exploring novel hybrid algorithms and their applications across various industries, further enhancing the accuracy and reliability of AI-driven forecasting models.

## 10. Critical Barriers to Widespread Adoption

As evidenced by the performance metrics compiled in Table 2, AI technologies are delivering tangible, quantifiable benefits across the dairy value chain, from animal health monitoring to supply chain optimization. Despite the compelling applications detailed throughout this review, the transition of AI from research laboratories and pilot projects to universal, reliable adoption on dairy farms is hampered by a series of profound and interconnected challenges. The issue extends far beyond the technical performance of algorithms to encompass fundamental practicalities of agricultural operation.

A primary obstacle is the pervasive problem of data scarcity and a lack of standardization. The development of robust, generalizable AI models is contingent upon access to vast, high-quality, and accurately labeled datasets. For many specific dairy-related parameters, such as nuanced disease manifestations or highly localized environmental impact metrics, such public datasets do not exist. This scarcity acts as a significant bottleneck, stifling innovation and making it difficult to independently validate and compare the performance of new models [81]. Furthermore, the computational demands and infrastructure costs associated with deploying real-time AI present a serious barrier, particularly for smallholder and remote farms. Solutions that rely on continuous video analysis, such as the CNN models used for body condition scoring [46] and estrus detection [47], or complex neural networks like the LSTMs employed for predicting disease onset [17], require substantial processing power and reliable high-speed internet connectivity, which is not a given in rural agricultural settings worldwide. This technological disparity risks creating a digital divide, where the benefits of AI are exclusively available to large-scale, well-capitalized operations, thereby exacerbating existing inequalities within the industry [21,85].

Even when the technical and infrastructural hurdles are overcome, adoption is not guaranteed. A significant impediment lies in the inherent difficulty in interpretation and lower trust in many advanced AI systems. The “black box” nature of sophisticated DL models like the hybrid CNNs and RNNs used for milk quality analysis [29] or the transformer models for pregnancy loss prediction [52] means that a farmer may receive an alert or a prediction without any clear understanding of the reasoning behind it. While techniques like SHAP (SHapley Additive exPlanations) offer a pathway to clarity, as demonstrated in the analysis of behavioral patterns for mastitis detection [10], their integration is not yet standard practice. Without this transparency, producers are often reluctant to act on the system’s recommendations, especially when those actions incur costs or disrupt routine. Therefore, the field of XAI is not merely an academic pursuit but a critical necessity for fostering the user confidence required for successful field deployment [10,36]. Finally, the challenge of seamless integration with existing farm workflows cannot be overstated. Dairy farming is characterized by demanding and often unpredictable daily routines. Any technology, no matter how advanced, that is cumbersome to use, requires excessive manual data entry, or disrupts established practices is likely to be rejected. For instance, while the MOOnitor device achieved 97% accuracy in activity tracking [82], its practical value is contingent on farmers comfortably adopting and maintaining the use of neck-mounted sensors on their entire herd. For AI to be truly embraced, it must be designed with the end-user in mind, offering intuitive interfaces and actionable insights that fit naturally into the operational rhythm of the farm [21,56].

## 11. Perspectives and Challenges

AI is increasingly transforming the dairy industry, contributing innovative solutions to long-standing challenges. Advances in this still-incipient branch of computer science are closing gaps in the way of workflow is approached in the food industry, unlocking opportunities to increase yield and profitability while softening the carbon footprint throughout the process. This new approach based on a circular economy opens the door to new business opportunities and prospects, which, given its novelty, requires special attention in order to detect deficiencies and apply the corresponding corrective measures.

A key perspective is the potential of AI to significantly enhance predictive accuracy. The development of hybrid AI methodologies, such as the integration of neural networks with meta-heuristic algorithms, has set a new standard for forecasting precision [54,93]. These approaches, tailored specifically for the unique requirements of perishable goods like dairy products, provide actionable insights that optimize inventory management and reduce waste. Incorporating diverse socio-economic and environmental factors, such as demographic data, economic indicators, and weather conditions, further enriches forecasting models, increasing their relevance and applicability [55].

Emerging technologies, including NLP for sentiment analysis, expand the scope of AI applications beyond traditional forecasting. For instance, sentiment analysis can capture consumer preferences and behaviors, providing real-time insights into market trends [97]. Coupled with subfields like computer vision and data mining, these advancements offer a multidimensional view of the dairy market, enhancing decision-making capabilities. Additionally, AI’s role in sustainability is noteworthy, as its integration can help minimize supply chain inefficiencies, reduce food waste, and support environmentally friendly production practices [93].

Despite these promising advancements, several challenges must be addressed to fully realize the potential of AI in this field. The reliance on high-quality and comprehensive datasets remains a critical barrier. Issues such as data sparsity, noise, and imbalances, while partially mitigated by techniques like the Synthetic Minority Oversampling Technique (SMOTE), can undermine the robustness of AI models [97]. Furthermore, the complexity of hybrid models, although offering superior accuracy, poses practical challenges related to computational demands and user accessibility [54].

Interpretability and transparency also remain pivotal concerns. While sophisticated AI systems often achieve high performance, their “black box” nature can limit stakeholder trust and hinder widespread adoption. Balancing the need for complex algorithms with the demand for understandable and transparent models is an ongoing priority [94].

The dynamic nature of the dairy market introduces additional challenges. Demand patterns are influenced by a myriad of factors, including seasonal variations, economic shifts, and policy changes. AI models must remain adaptable to these evolving conditions, necessitating frequent updates and validations [92]. Moreover, regional differences in consumer behavior and production practices complicate the scalability of AI solutions, requiring tailored approaches for different contexts [93].

Integrating AI-driven methods with traditional economic models and established industry practices is another critical area. While AI can capture intricate patterns in large datasets, conventional models often provide a broader theoretical framework, especially in incorporating demographic effects [55,97]. Harmonizing these approaches is essential for generating comprehensive and reliable forecasts.

Ethical considerations further complicate the landscape. The use of AI in sentiment analysis raises concerns about data privacy and potential biases in decision-making. Ensuring ethical practices in data collection, processing, and analysis is crucial for maintaining public trust and regulatory compliance [97].

AI-driven demand forecasting presents transformative opportunities for the dairy industry, enabling more accurate predictions, efficient resource allocation, and sustainable practices. Future research should prioritize the development of user-friendly and interpretable models, the creation of robust datasets reflective of diverse market conditions, and the ethical implementation of AI technologies. Collaboration among AI researchers, industry professionals, and policymakers will be vital to overcoming current challenges and unlocking the full potential of AI in demand forecasting.

## 12. Discussion on the Implementation of Artificial Intelligence (AI) in the Dairy Industry

The evidence presented in this review establishes AI as a powerful catalyst for transforming dairy production, enabling a shift from traditional practices to a data-driven and sustainable paradigm. Despite rapid innovation in specialized areas like health monitoring and demand forecasting, the full potential of AI will only be realized by moving beyond these standalone applications. The principal challenge is now practical: creating fully integrated systems that are interoperable and implementable on working farms, regardless of their scale.

A critical finding of this synthesis is that the potential of AI is limited by data silos. Most current applications operate in isolation, whereas their value would be multiplied through integration. For instance, an AI system that predicts a mastitis infection should be directly linked to systems for milk quality assessment, production forecasting, and supply chain logistics. Currently, such interconnected solutions are not the norm. Future progress depends on designing systems with interoperability as a core principle, leveraging architectures like the data-mesh concept exemplified by CowMesh [80] to create a unified data infrastructure for the entire industry.

Furthermore, a significant implementation gap persists between experimental results and practical on-farm application. While reported accuracies of models are often impressive [29,82], their real-world value is dictated by explainability, accessibility, and data integrity. Farmers must trust the technology to act on its insights, necessitating a greater focus on XAI techniques that demystify algorithmic decisions, as demonstrated by Shi et al. [10]. Simultaneously, the development of low-cost, edge-computing solutions [15,27,47] is paramount to ensuring these advancements benefit not only large-scale operations but also the small and medium-scale farms that are vital to global dairy production. Underpinning all of this is the unresolved challenge of data quality and standardization; the industry must collectively champion the creation of open, benchmark datasets, like CowScreeningDB for lameness [81], to ensure models are built on a foundation of reliable and consistent information.

Looking forward, the research trajectory must evolve to address these synthesis and implementation challenges. Priority should be given to developing hybrid models that marry the predictive power of DL with the interpretability of domain-specific knowledge. A particularly promising frontier lies in deploying AI for direct, real-time measurement of sustainability metrics, such as individual methane emissions, moving beyond optimization to precise quantification [13,23]. Ultimately, the most critical research direction may be human-centric AI design, which focuses on creating intuitive interfaces and decision-support tools that integrate gracefully into the demanding daily routines of farmers.

In conclusion, the future of AI in dairy is not merely a linear path of improving algorithmic performance, but a journey towards creating smarter, more connected, and more equitable systems. By focusing on integration, explainability, and accessibility, the industry can harness AI to build a future that is not only more productive and profitable but also more transparent, sustainable, and attentive to the welfare of animals, the expectations of consumers, and the health of the environment.

## 13. Conclusions

Artificial intelligence is poised to fundamentally transform the dairy industry, driving advancements in productivity, sustainability, and animal welfare. Its applications span the entire production chain, from enabling real-time health monitoring and precise feeding strategies to optimizing milk quality, streamlining the production of derivatives, and enhancing supply chain logistics through accurate demand forecasting. Furthermore, AI provides critical tools for measuring and mitigating the environmental footprint of dairy farming. Despite this immense potential, widespread adoption hinges on overcoming significant barriers, including data standardization, computational costs, and the seamless integration of these technologies into practical farm management. Addressing these challenges is essential to fully leverage AI for building a more efficient, transparent, and resilient dairy sector capable of meeting global demands.

## Figures and Tables

**Table 1:** Examples of application of artificial intelligence (AI) and machine learning (ML) applications in areas of dairy production and the dairy industry.

Name	Algorithm Type	Area of Implementation	Results	Authors
Random Forest (RF) algorithm	ML (Machine learning)	Animal health	Early detection of mastitis using wearable sensors and behavioral analysis	Shi et al. [10]
ThinkDairy Data-Mining Hub	ML		Enhanced detection of heat stress and subclinical mastitis through K-Nearest Neighbors (KNN)	Nogoy et al. [25]
Long Short-Term Memory (LSTM) models	DL (Deep learning)		Predicted anaplasmosis onset 3–5 days before clinical symptoms appeared	Teixeira et al. [26]
YOLO v8 for thermal imaging	DL		94.58% accuracy in detecting dairy cow respiratory rates	Zhao et al. [27]
SHAP Explainable AI (XAI)	Other (XAI)		Identified combined behavioral patterns linked to health issues	Shi et al. [10]
Multilayer Perceptron (MLP)	DL		Detected subclinical ketosis in dairy cows, showcasing effectiveness in managing herd health	Bauer and Jagusiak [28]
Regression Tree Models	ML	Nutrition and Feed Efficiency	Predicted impact of dietary interventions on milk composition and yield; improved resource utilization while maintaining milk quality	Monteiro et al. [16]
Back Propagation Neural Networks (BPNN) optimized with Genetic Algorithms (GA)	DL (BPNN)/Other (GA)	Milk production	Accurate prediction of milk yield based on environmental and physiological factors	Monteiro et al. [16]
Convolutional Neural Networks (CNNs) with Recurrent Neural Networks (RNNs)	DL	Milk quality	Achieved 98.03% accuracy in milk quality prediction	Mhapsekar et al. [29]
Adaptive Neuro-Fuzzy Inference Systems (ANFIS)	Other (Neuro-fuzzy)	Milk composition	Real-time prediction of milk moisture and fat content with high precision	Alsaedi et al. [14]
Edge-AI	DL	Milk adulteration detection	Achieved 94.87% accuracy in classifying adulterants using Fourier Transformed Infrared data	Mhapsekar et al. [15]
Biometric and Environmental Sensor Technology (BEST)	ML	Quality Monitoring & Safety	Enhanced milk sample categorization, supporting HACCP protocols	Dragone et al. [30]
Hybrid Machine Learning (Deep and Shallow Learning)	DL	Dairy derivatives	Automated ripeness detection in cheese with 99.10% accuracy	Zedda et al. [18]
MLP	DL	Environmental sustainability	Optimized factory productivity with near-perfect accuracy (R^2^ = 0.99984)	Oztuna Taner et al. [20]
Ant Colony Optimization (ACO)	Other (Optimization)	Logistics	Optimized milk collection routes, reducing emissions and costs	Karouani et al. [17]
Facial Action Coding System (FACS)	DL	Consumer preference	Evaluated infant responses to yogurt, guiding product development	Gülsen et al. [31]

HACCP: hazard analysis and critical control points.

**Table 2:** Quantitative Improvements from AI Applications in the Dairy Industry.

Area of Application	Specific AI Technology	Task	Key Performance Metric	Result	Reference
Animal Health	YOLO v8 (DL)	Respiratory rate detection	Accuracy	94.58%	Zhao et al. [27]
Animal Health	Long Short-Term Memory (LSTM)	Anaplasmosis prediction	Early Detection Lead Time	3–5 days before clinical symptoms	Teixeira et al. [26]
Animal Health	Multilayer Perceptron (MLP)	Subclinical ketosis detection	Effectiveness	Successfully detected condition	Bauer and Jagusiak [28]
Milk Quality	CNNs + RNNs (HyBDL)	Milk Quality Analysis	Accuracy/Mean Squared Error (MSE)	98.03% accuracy (with lower MSE)	Mhapsekar et al. [29]
Milk Quality	Edge-AI (CNN)	Milk adulteration detection	Accuracy	94.87%	Mhapsekar et al. [15]
Dairy Derivatives	Hybrid ML (Deep & Shallow)	Cheese ripeness detection	Accuracy/F-measure	99.10% accuracy (F-measure: 0.991)	Zedda et al. [18]
Production & Environment	Multilayer Perceptron (MLP)	Factory productivity prediction	Coefficient of Determination (R^2^)	R^2^ = 0.99984 (Near-perfect prediction)	Oztuna Taner et al. [20]
Logistics	Ant Colony Optimization (ACO)	Milk collection route optimization	Outcome	Reduced emissions and costs	Karouani et al. [17]
Demand Forecasting	MLP + Runner-Root Algorithm (RRA)	Dairy product demand prediction	Coefficient of Determination (R^2^)	R^2^ = 98.19%	Goli et al. [54]
Demand Forecasting	Statistical tests + MLP	Dairy product demand prediction	Error Reduction	Forecast errors reduced by 1.8 times	Goli et al. [55]

**Table 3:** AI models applied to dairy yield prediction and optimization.

Model	Input Variables	Reported Performance	Application Scope	Reference
BPNN	Lactation stage, body weight, feed intake	Dry matter intake (standard variables + microbiome) R^2^ = 0.89; Milk fat efficiency (combined) R^2^ = 0.92; Milk protein efficiency (combined) R^2^ = 0.84	Individual yield estimation	Monteiro et al. [16]
ACO-BPNN	Route data + herd production	Cost reduction by 18%	Farm logistics optimization	Karouani et al. [17]
Multilayer Perceptron (MLP)	Nine product types	R^2^: 0.99984, MSE: 4.02 × 10^−6^	Factory productivity prediction	Oztuna Taner et al. [20]
Predictive analytics	Large historical datasets	Enhanced efficiency and production outcomes (does not specify dairy production)	Yield optimization through data-driven decisions	Martin et al. [59]
IoT & AI integration	Real-time sensor data	84% reduction in transaction costs, increased milk production, and overall operational efficiency.	Smart dairy farming systems	Akbar et al. [60]
Deep Learning Models	Agricultural and livestock data	Efficient yield prediction and management (does not specify dairy production)	Automation and sustainable farming practices	Rane et al. [61]

**Table 4:** Representative AI models for milk quality and safety assessment.

Model	Analytical Target	Accuracy	Sample Type	Key Contribution	Reference
ANFIS	Fat and protein content	R^2^ = 0.93	Raw milk	First fuzzy inference system for milk components	Alsaedi et al. [14]
CNN	Adulteration detection	98.5%	NIR spectra	Non-destructive online testing	Mhapsekar et al. [15]
HyBDL	Bacterial contamination	99.2%	UHT milk	Hybrid deep-learning framework	Mhapsekar et al. [29]
GRU-ResNet	Microbial safety classification	F1 = 0.97	Pasteurized milk	Time-series–image fusion model	Tolba et al. [65]
XGBoost & Regression Models	Protein and fat content prediction	Not specified	Multi-spectral milk samples	Real-time quality control using multi-spectral sensing and edge computing	Durgun et al. [67]
Distance-Weighted KNN (DW-KNN)	Milk quality classification	99.53%	Milk samples for quality detection	Significant improvement over standard KNN for high-precision classification	Samad et al. [68]
Multilayer Perceptron (MLP)	Factory productivity and shelf-life	R^2^ = 0.99984	Factory production data	Enhanced processing efficiency and sustainable shelf-life extension	Taner and Çolak [20]
MLP Neural Networks	Milk sample categorization	Enhanced accuracy	Biometric and environmental sensor data	HACCP protocol support and traceability monitoring	Dragone et al. [30]

**Table 5:** Artificial intelligence (AI) for improving the quality and sensory experience of dairy products.

AI Technology	Task	Key Performance Metric	Result	Reference
Hybrid ML (Deep & Shallow)	Cheese ripeness detection	Accuracy/F-measure	99.10% accuracy (F-measure: 0.991)	Zedda et al. [18]
Computer Vision + ML	Cheese ripening assessment	Classification Accuracy	98% classification accuracy	Loddo et al. [72]
GA + PSO + SVM	Yogurt sensory optimization	Consumer Preference Alignment	Identified consumer-preferred sensory features	Bi et al. [19]
Facial Action Coding System (FACS)	Infant emotional response to yogurt	Emotion Analysis	Identified negative reactions (disgust, fear) in breastfed infants	Gülsen et al. [31]
Neural Networks (FaceReader)	Predict purchase intent from ads	Prediction Accuracy	90–91% accuracy in predicting purchase intent	Tzafilkou et al. [74]
Computer Vision + Temporal Data	Curd cutting time determination	Process Optimization	Enabled immediate adjustments, enhancing consistency	Loddo et al. [73]
XGBoost Model	Real-time milk quality control using multi-spectral sensing and edge computing	R^2^ > 0.85 for protein and fat prediction	Enables rapid on-site detection and enhances milk quality control efficiency	Durgun (2024) [67]
K-Nearest Neighbors (KNN) & Distance-Weighted KNN (DW-KNN)	Milk quality detection	Accuracy: 99.53% (DW-KNN) vs. 98.58% (KNN)	Distance weighting improves classification precision in milk quality assessment	Samad et al. [68]
Multilayer Perceptron (MLP) Artificial Neural Network	Productivity analysis in dairy factories	Process optimization and shelf-life extension	Models data input–output relationships to enhance processing sustainability	Taner & Çolak [20]
Flavor and Sensory Experience Enhancement (AI-assisted)	Identification of key flavor compounds in fermented milk	Odor Activity Value (OAV)	Identified linalool and mushroom alcohol as main aroma and off-flavor drivers	Chi et al. [75]
Functional Ingredient Optimization via AI Modeling	Silkworm pupae peptide effects on yogurt fermentation	Physicochemical and textural metrics	Improved firmness, cohesiveness, acidification profile, WHC; altered flavor balance	Wang et al. [76]
Dairy Product Processing Innovations (AI-integrated analysis)	Impact of milk types, probiotics, additives on yogurt quality	Physicochemical and sensory attributes	Improved flavor, gel stability, and consumer acceptance	Farag et al. [77]
Instrumental–Sensory Integration Models	Probiotic yogurt with almond milk: correlation of instrumental and sensory data	Color, texture, and sensory metrics	Multivariate analysis improved alignment between instrumental and sensory perception	Yilmaz-Ersan & Topcuoglu [78]

**Table 6:** AI-Based Dairy Databases and Data Integration Platforms.

Database/System	Data Types Integrated	AI/ML Components	Key Application	Key Contribution	Reference
CowMesh	Activity monitors, herd records, management systems, milking logs	ML algorithms for health prediction	Comprehensive farm management	Data-mesh architecture for unifying disparate dairy data sources	Pakrashi et al. [80]
CowScreeningDB	IMU sensor data, clinician lameness scores	Deep learning models	Lameness detection	Public multi-sensor database with FAIR principles for reproducible research	Ismail et al. [81]
MOOnitor	Temperature, GPS, accelerometer data	XGBoost, Random Forest	Activity classification and health monitoring	Integrated IoT device with 97% activity classification accuracy	Dutta et al. [86]
Dairy Brain Concept	Electronic health records, production data, environmental sensors	Predictive analytics	Farm optimization	Conceptual framework for integrated data analytics in dairy operations	Cabrera & Fadul-Pacheco [79]
Sustainability Database	Emissions data, energy usage, feed efficiency metrics	Big data analytics	Environmental impact assessment	Benchmarking standards for net-zero emissions tracking	Neethirajan et al. [13]

**Table 7:** Quantitative Summary of AI Applications in Environmental Impact Assessment.

AI Technology	Task	Key Performance Metric	Result	Reference
AI & Big Data Analytics (Conceptual)	GHG emissions reduction/Net-zero roadmap	Benchmarking/Transparency	Enables benchmarking and supply chain transparency	Neethirajan [13]
Fault Detection & Diagnosis (FDD) + Transfer Learning	Refrigeration system efficiency	Energy Savings	Improved energy efficiency, addressed data scarcity	Lee et al. [84]
Machine Learning Algorithms	Manure temperature prediction	Prediction Accuracy	Improved accuracy of temperature predictions for nutrient/emissions management	Genedy et al. [83]
Artificial Neural Networks (ANN)	Transport system optimization	Operational Efficiency/Cost	Reduced operational costs and emissions	Świderski et al. [88]
Industry 4.0 Tech (AI, IoT, Blockchain)	Waste reduction and traceability	Resource Efficiency/Quality	Improved traceability, reduced waste, optimized supply chains	Hassoun et al. [85]

**Table 8:** Summary of AI Applications in Demand Prediction.

AI Technology	Task	Key Performance Metric	Result	Reference
MLP + Runner-Root Algorithm (RRA)	Dairy product demand prediction	Coefficient of Determination (R^2^)	R^2^ = 98.19%	Goli et al. [54]
Statistical tests + MLP + GWO/CA	Dairy product demand prediction	Error Reduction	Forecast errors reduced by 1.8 times	Goli et al. [93]
SVR + MLP + ANFIS + IWO/PSO	Dairy product demand prediction	Predictive Accuracy	Improved predictive accuracy vs. conventional methods	Goli et al. [55]
RBF Neural Networks + GA	Sales forecasting for short shelf-life products	Forecasting Accuracy	Outperformed traditional methods	Doganis et al. [92]
NLP + Data Balancing (SMOTE)	Sentiment analysis for dairy products	Model Performance/Classification	Improved model performance for consumer insight	da Silva Nogueira et al. [97]
LSTM (Long Short-Term Memory)	Forecasting dairy product demand across 5 production plants	Mean Absolute Scaled Error (MASE) < 1.0 for 36/40 series	Outperformed ARIMA in 28/40 product series; achieved ~70% overall winner share; monthly data produced better accuracy than weekly	Vithitsoontorn & Chongstitvatana [90]
ARIMA (AutoRegressive Integrated Moving Average)	Baseline statistical model for demand forecasting	MASE > 1.0 in most cases compared with LSTM	Performed better on stable demand series with minimal fluctuation	Vithitsoontorn & Chongstitvatana [90]
Feed-Forward Neural Network (NN)	Regional dairy product demand prediction (Amul butter)	Not specified	Demonstrated feasibility of neural networks for short-term dairy demand forecasting; used demographic and infrastructure variables	Vimal Kumar et al. [91]

## Data Availability

No new data were created or analyzed in this study. Data sharing is not applicable to this article.
